# The Role of Green Human Resource Practices in Fostering Green Corporate Social Responsibility

**DOI:** 10.3389/fpsyg.2022.792343

**Published:** 2022-04-15

**Authors:** Rizwana Hameed, Asif Mahmood, Muhammad Shoaib

**Affiliations:** ^1^Institute of Business and Management, University of Engineering and Technology, Lahore, Pakistan; ^2^Department of Business Studies, Namal Institute, Mianwali, Pakistan; ^3^Department of Computer Science, University of Engineering and Technology, Lahore, Pakistan

**Keywords:** PEPC, CSR, IT capabilities, GHRM practices, PEB

## Abstract

This study develops a conceptual framework and investigates green human resource practices (GHRM)—green recruitment and selection, green training and development, and green reward and compensation? effects on pro-environmental psychological climate and pro-environmental behavior, which cause green corporate social responsibility (GCSR). We employ information technology (IT) capabilities as a moderator between the GHRM and pro-environmental behavior. It applies a convenience sampling technique and survey questionnaire to collect data from 388 employees at CPEC projects. Results demonstrate that GHRM positively influences pro-environmental psychological climate and pro-environmental behavior that significantly develops GCSR. IT capabilities significantly moderate the relationships between GHRM and pro-environmental behavior. The study findings add to the body of green HRM practices, strategic management, and information processing and policy makers better postulate, align, and exercise their green HRM practices for its synergetic effects for green CSR and sustainability. We also acknowledge some limitations and provide future directions.

## Introduction

The emergence of global ecological challenges has compelled hard organizations to adopt eco-friendly policies in order to mitigate the negative effects of wasteful resource usage and environmental degradation and still be green (Afsar and Umrani, 2020). Air pollution in cities, unnatural weather changes, water scarcity, and biodiversity loss as a result of severe environmental degradation, all of which put the globe in grave peril, are major issues (Malik et al., 2020). Human behaviors, enormous technical innovation, and a lack of proactive corporate social responsibility (CSR) approaches for long-term performance are all factors that contribute to these issues. Geopolitical problems, such as the emergence of anti-globalization sentiment around the world, the flood of immigrants and refugees, and the resulting xenophobic backlash in several countries, create new challenges for organizations (Stahl et al., 2020).

Along these lines, the 2008 financial crisis, which exposed corporate financial misdeeds and failures, erased billions of dollars in market value, causing our modern organizations and economic models to fail (Waldman and Siegel, 2008). Apple, Coca-Cola, and Walmart, for example, have all been involved in environmental and social problems (Torres et al., 2012). These considerations lead us to believe that non-ecological business activities create significant environmental challenges both inside and outside of organizations, necessitating CSR.

As a result, a clear link between a company’s CSR programmes and its internal and external stakeholders is mandatory (Ramkissoon et al., 2020). Top management frequently initiates the formation and growth of a CSR-focused business culture, which gradually changes employee attitudes and performance around CSR practices (Ramkissoon et al., 2020). Furthermore, once an individual senses a strategic alignment between his or her individual identity and the characteristics of the company (Ramkissoon et al., 2020), they are far more inclined to work hard to accomplish the company’s CSR goal (Sowamber and Ramkissoon, 2019), which is why authors believe that green human resource practices (HRM) practises play such an important role in creating a green climate and improving employee green behavior. Accordingly, Islam et al. (2019), underlined the critical relevance of the green aspects of HRM within the organizations? green human resource management (GHRM). In response, we are taking all these into consideration and argue that CSR continues to grow. The body of HRM refers that “HRM function is uniquely positioned to assist in both developing and implementing [CSR and] sustainability strategy” help to combat such challenges (Cohen et al., 2012).

The significance of green HRM practises in sustainability performance, has been emphasized by a few researchers like (Pham et al., 2019; Yusliza et al., 2019; Aboramadan, 2020; Darvishmotevali and Altinay, 2022). The selection of HRM procedures and practices have assumed a critical part in managing these difficulties and conveying CSR activities beyond publicity (Stahl et al., 2020). Consequently, the basic test for enterprises is to completely incorporate CSR into their procedures, plans of action, and working cycles to construct societies that help the vital change of mentalities and practices. However, it has been ignored to define the CSR phenomenon in the global context. In light of varied stakeholders and non-administrative firms, which influence from diverse natural and social settings in international trades (Freitas et al., 2020).

Both the scholars and professional literary studies indicate that organizations may profit commercially from fusing obligation and sustainability standards into their systems, strategies, and fundamental corporate practices (Stahl et al., 2020). Nevertheless, it has been insufficiently investigated and HRM experts are not as of now acknowledged as accomplices in impacting CSR procedures, nor is HRM a key implementer of CSR activities and projects (Cohen et al., 2012). Portrayals of HRM as a component of CSR investigate what different GHRM activities mean for CSR, and how CSR energizes practices to defeat the impacts of non-ecological challenges.

The existing literature contended that persuading workers to display an uplifting disposition and conduct in lessening firms’ ecological impression and improving sustainable strategic policies. For instance, behavioral studies distinguished that HRM is diffused *via* innumerable essential mechanisms (Stahl et al., 2020) which do not directly affect employee behavior. It has been accredited for a long time by environmental psychologists that pro-environmental behavior (PEB) is affected by contextual aspects. PEBs allude to any quantifiable ecological practices assisting firms with turning out to be naturally sustainable (Chaudhary, 2020)with psychological climate—“workers’ impression of workplace qualities or firms” which pondered huge context-oriented effect on firm behavior. Still, regardless of rising academic interest in connecting green HRM with representatives’ work environment green conduct like PEBs, CSR, and sustainability (Saeed et al., 2019; Stahl et al., 2020) relationships have not been sufficiently examined in the existing body of knowledge.

Based on the aforementioned contributions, we contend that green CSR—the waste-decrease exercise of the organization to exploit the productivity of their inputs and limit the methods for contrarily affecting the people in the future is the only solution to such grave issues. In response, we followed the work of Saeed et al. (2019) who acknowledge that to achieve the organizational green goals the firms should endorse green HRM (GHRM) to foster and prompt worker eco-friendly performance. Technological advances are being driven essentially by solid demands from green HR experts for an upgrade in speed, viability, and cost control which results in greater profitability and sustainability of the organization (Daneshvar Kakhki and Gargeya, 2019; Vijayalakshmi and Vidya, 2020). Research suggested that IT represents 2% of worldwide CO2 discharges, which is proportionate to the sum produced by aviation businesses (Goasduff and Forsling, 2007). Keeping in view the above discussion the underlying study is aimed at how GHRM practices is going to shape and strengthen green CSR practices.

We conceptualized and used the stakeholder theory as a theoretical foundation. This theory postulates that a firm has numerous partners who have interests in, and whose interests are affected by the company’s practices (Freeman, 1984). Thus, the present study fills this gap by presenting and validating a research model to examine the effects of green HRM practices (i.e., green recruitment and selection, green training and development, and green reward and compensation) in developing a pro-environmental psychological climate (PEPC) and PEB. We investigate the underlying mediating mechanism of PEPC and PEB between GHRM practices and GCSR. This work also examines the moderating effect of IT capabilities between GHRM practices and PEB. To answer the research questions, our findings add to the literature regarding green HRM practices by division of the GHRM into three measurements. Moreover, the findings additionally focus on the IT capabilities by empirically and hypothetically showing how IT capabilities influence the green conduct of workers. It contributes to the body of knowledge in the fields of strategic management by reviewing the literature and validating the mediation effect of green behavior and psychological climate. It helps the managers and policymakers in developing countries to better postulate, align and exercise their green HRM practices for its synergetic effects for green CSR and sustainability.

## Theoretical Background and Hypotheses Formulation

### Stakeholder’s Theory

The stakeholder theory is a popular corporate philosophy and management theory that advocates efficient, pragmatic, and ethical approaches to organize and manage organizational concerns in a variety of scenarios (Freeman, 2010; Harrison et al., 2015). Such theories, from a global context, help to uncover economic and societal concerns by aiding organizations’ strategic decisions (Waheed and Zhang, 2020).

The stakeholder theory has its underlying foundations during the 1960s organizational literature, albeit the theory’ formalization is regularly credited to Freeman by Laplume et al. (2008). As per stakeholder theory *“managers should focus on any group or person who can influence or is influenced by the firm’s objective, since that group may forestall [the firm’s] achievements.”* This theory tends to talk about morals and standards in organizational management. Stakeholders hold authority, to affect an organization’s performance and sustainability from alternate points of view in inconsistent measures (Laplume et al., 2008).

Since, stakeholder theory, as a “theory of organizations,” has aided in the development of a relational organizational model (Damak-Ayadi and Pesqueux, 2005), as it starts with the premise that values are both implicit and explicit in doing business. It invites management to express a shared understanding of the value they provide and what binds the company’s key stakeholders together. The stakeholder theory unequivocally or obliquely encompasses a theory of three distinct sorts —descriptive/empirical, instrumental, and normative. In this context, the descriptive aspect with the question for instance “What does happen?,” resembles the inductive technique, which attempts to infer general rules and conclusions from examining individual preferences (Steurer, 2006). Descriptive/empirical formulations of the theory are planned to portray as well as clarify how firms or their administrators carry on. Therefore, the role of GHRM practices (green recruitment and selection, green training and development, and green reward and compensation) are very vital to better comprehend the description of a job by the personnel. The right skill set earned on the job after training would lead the mangers to be green and do his/her job more efficiently and effectively. The green human resource practices are intended to make a labor force, which comprehends and advances green conduct in the firm (Hameed et al., 2020) as the adequacy of any essential method is subjected to the accessibility and ability of its people (Jackson and Seo, 2010).

The instrumental aspect encompasses both the normative/deductive and descriptive/inductive approaches; its distinguishing trait is that it emphasizes causality by connecting means and goals by the “What would happen if?” perspective (Jones, 1980). Instrumental describes the demands and how to satisfy the internal and external stakeholders, how would prosper financially, socially, and environmentally in return to generate value for all the concerned parties. It is accepted that the manifestation of a perceived green environment because of the systematization of the corporate ecological approach sets up that the green conduct is worthwhile for the firm and workers. Consequently, the workers gain from both corporate ecological procedures and observe green environment to carry on in an ecologically responsible manner. Accordingly, the positive pro-environmental psychological climate is indispensable to engender green behavior among the employees.

Last but not the least, the normative part resembles the deductive technique, which tries to apply common rules to particular instances (typically based on ethical considerations) with questions like “What should happen?” From a normative standpoint, the supervisors’ behavior must be ethically respectable. Organizations are well-equipped to respond to pressures from stakeholders like precise prerogatives of stakeholders developed on the basis of their explicit benefits and requirements and are bound to enjoy greater performance. For this, Orlitzky et al. (2003), demonstrate that organizations are equipped appropriately to deal with their stakeholders’ experience, which causes greater financial outcomes.

**FIGURE 1:**
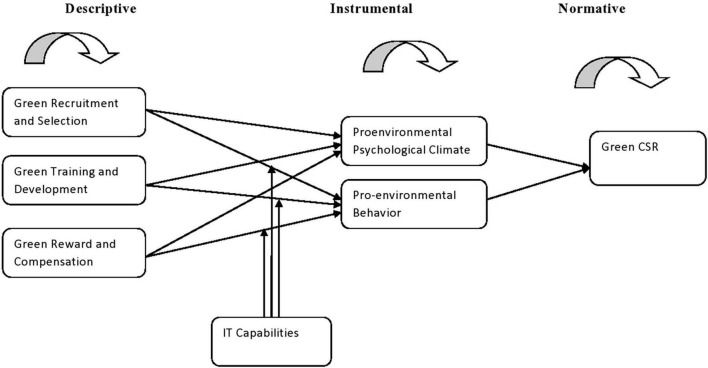
The descriptive, instrumental, and normative perspective of the stakeholder theory.

As indicated by Sroufe (2017), the demands from the stakeholders and intended activities will appropriately guide and help the executives in identification, comprehension, and evolving both the internal and external environment of businesses. It eventually established a strong foundation for the strategic planning of the firm while attaining sustainable growth. It has just been uncovered that the demands from the stakeholders propel organizations to mull over societal, ecological, and wellbeing and security issues and challenges. Organizations can address stakeholders’ prerequisites by and large through unequivocally concentrating on the progress and advancement of significant performance indicators alongside the valuation of each indicator on priority. The core concern of firms’ CSR practices is to connect with stakeholders and meet their valid demands and desires, and the role of GHRM practices are vital and that is why stakeholder’s theory provides relevance in this context. The following conceptual model describes the combination of all respective perspectives in Figure 1.

### Hypotheses Formulation

#### Green Recruitment and Selection and Pro-environmental Psychological Climate and Pro-environmental Behavior

Green recruitment and selection (GRS) are indispensable to attract and build effective human capital in an organization. GRS is *referred to as “a process of recruiting and selecting candidates who are sensitive to environmental issues and willing to commit to environmental performance”* (Tang et al., 2018). Many researchers like (Islam et al., 2019; Saeed et al., 2019) view that the potential job seekers comprehend that if the organization deals well with their existing workers and society, certainly they would be likewise treated, On the other hand, if this perception went wrong, the potential candidates may lose attraction toward the respective firm. GRS is amid at dealing with a large reservoir of the potential candidate among which the most skilled ones got hired. The employees with green conscientiousness would pave the path to build strong culture and climate of proactive self-responsibility (Tang et al., 2018). In a similar vein, Wehrmeyer and Parker (1996) and Zoogah (2011) evaluate employees appropriately before their final selection. Since pro-environmental work climate projected workers both an independent and meticulous reassurance to get involved in green (Saeed et al., 2019). Therefore, employees anticipate the work environment to be inspiring where a pro-environmental psychological environment refers *to “an employee’s shared perceptions and interpretations of organizational policies, the procedures that translate policies into guidelines, and the practices expected and rewarded by the organization”* (Chaudhary, 2020). It means the right candidates after this GRS process are capable enough to build their perceptions and behave accordingly while maintaining their positive perception for the respective employers and job openings (del Brío et al., 2007). Accordingly, we argue that the selection of employees with green consciousness would help the organization in building a strong climate to build future strategies in pursuance of better environmental performance. It refers to those representatives who are sensitive to environmental issues and keen to benefit firm to pursue firm’s green objectives that is in line with the discussion made above.

The worker’s favorable green conduct basically adds to encourage ecological performance since employee green behaviors is defined as the *“willingness to engage in pro-environmental activities”(Saeed et al., 2019)*. The newly selected employees, once joined, would build favor dealt with green recruitment and selection in diverse situations (Tang et al., 2018). Taking into account the preceding debate, we posit that the hiring of the right employees through green recruitment and selection practice helps organization to build green environment. This green climate thus motivates the employees to engage, behave, and refer the environmental practices to achieve organizational green goals. Therefore, we proposed that.

H1: *Green recruitment and selection positively associated with (a) pro-environmental psychological climate and (b) pro-environmental behavior.*

#### Green Training and Development, Pro-environmental Psychological Climate, and Pro-environmental Behavior

Green training enhances skills, knowledge, and develops sustainable behavior of employees by providing training about environmental sustainability (Zoogah, 2011). Researchers like (Pinzone et al., 2019; Saeed et al., 2019; Elahi et al., 2020; Hameed et al., 2020) conceptualized green training and development (GTD) as “*a system of activities that motivate employees to learn environment protection skills and pay attention to environmental issues, which is key in accomplishing environmental objectives.*” GTD plans to improve workers’ mindfulness and information on ecological issues, fabricate inspirational mentality, assume a positive approach to tackle ecological apprehensions along with generating capabilities to monitor energy consumption and waste reduction (Zoogah, 2011). To change the attitude of the employees, organization should conduct seminars and workshops on environmental education. Environment-oriented training sessions can enhance workers’ mindfulness, familiarity, and skills to build a green climate. Based on the above discussion, we infer that GTD assembles an environment that urges all representatives to get associated with ecological activities. Likewise, employees after learning the requisite skills through GTD would create a congenial work environment. Since, the presence of such green climate pulls employees to sense their responsibility toward the accomplishment of the organizational green goals.

The firm’s apprehension for the climate alongside its endeavors to contribute toward greening looks for representatives to lock in pro-environmental behaviors (Renwick et al., 2013). Employee behaviors can likewise be formed through intervening mechanisms that usually target persuasive approaches to relate to knowledge, training, growth, and structural change (Jabbour et al., 2013) the positive causal association between green training and green behavior of employees have confirmed (Teixeira et al., 2012). Investigations on training have, indeed, featured that it might likewise result in expanding workload time pressure, and stress. Green training, on the other hand, is also found in an inverse relationship with green behavior (Yuriev et al., 2018). Given the above argument, it is clear that a representative’s willingness to initiate and carry out ecofriendly initiatives is inextricably linked to the sustainable workplace reflected through green HR practices.

Accordingly, “we contend that training will uncover the workers engaged with a super task to the significant environmental knowledge that could trigger their anxiety for the climate.” Thus, we propose that:

H2: *Green training and development are positively associated with (a) pro-environmental psychological climate and (b) pro-environmental behavior.*

#### Green Reward and Compensation Pro-environmental Psychological Climate and Pro-environmental Behavior

Among the GHRM system, green benefits and compensation system encourages those employees who meet the organization’s environmental standards (Jabbour et al., 2013). The extant literature signifies green HRM practices and their contribution toward strengthening work environments (Islam et al., 2019; Hameed et al., 2020). Green award and remuneration are the arrangements of monetary and non-monetary prizes, targeting pulling in, holding, and persuading workers to contribute toward the company’s ecological objectives (Jabbour et al., 2013). It has been proposed by many experts that the rewards urge workers to actively participate in organizational green practices (Daily and Huang, 2001; Govindarajulu and Daily, 2004; Jackson et al., 2011). However, it is hard to design measures for reward in order to precisely and equitably reward workers on the basis of ecological performance (Fernández et al., 2003). As per the study of Chaudhary (2020) pro-environmental work climate projected workers both an independent and meticulous inspiration to get involved in PEB, workers get signs of their surroundings after determining whether to exhibit green behavior to get compensated (monetary/non-monetary) or not. Hence, inferring from the above debate, the employees working in different organizations under the CPEC need to be motivated to earn these monetary and non-monetary rewards as per their contribution toward ecological performance.

The green reward and remuneration framework intend to align green practices and behaviors embraced inside the firm while the performance assessment framework affirms the appraisal of green practices (Jabbour et al., 2013). Infract, a green reward approach guarantees that the incentives and benefits are linked with this assessment (Renwick et al., 2013). Nishii et al. (2008) proclaim that workers’ work perspectives and practices are dependable on firm GHRM practices. Similarly, green HRM inside a firm influences the working environment and pro-environmental behavior among workers (Dumont et al., 2017). Thus, if greening is fused in firm HR practices and policies, workers would practice that behavior that resonates with the firm’s green strategies. At the point when a firm presents rewards for state-of-the-art ecological initiative/performance, employees turn out to be inspired to contribute to green activities. It means employee’s green initiatives got acknowledged within a firm. Based on the above arguments, we proposed that:

H3: *Green reward and compensation are positively associated with (a) pro-environmental psychological climate and (b) pro-environmental behavior.*

#### Pro-environmental Psychological Climate and Green Corporate Social Responsibility

Even though a person’s conduct is resolved to an enormous degree by their own qualities, it is likewise formed by the milieu in which they work. Environment development is reinforced through the level of organizational or formal accentuation for specific strategies (Kuenzi and Schminke, 2009). In like manner, the green climate might impact the association between the green climate and their green behaviors. It employs that the role of organizational culture and climate are critical. Thus, organizations work under green authoritative culture perceive to fathom issues, and create procedures that extraordinarily assist the organization with exploring through the ecological qualities (McCullough et al., 2016). On the other hand, individuals are regularly propelled to actualize pro-environmental behaviors, yet these attractive practices might be obliged when relevant support is inadequate in each setting.

Henceforth, the pro-environmental psychological climate implies a plausible and significant strategy for specialists to oversee and secure the climate. However, few investigations have been directed to confirm the part of pro-environmental climate (Wang et al., 2020). An examination conducted by Chou (2014); Paillé and Raineri (2015) shows that individual and authoritative variables consolidate in a comprehensive system that impacts worker commitment in extra-role behavior. Considering the extant literature, to practice green CSR, the green climate is thus mandated among organizations working under CPEC. Therefore, we hypnotized as:

H4: *Pro-environmental psychological climate positively influences green CSR.*

#### Pro-environmental Behavior and Green Corporate Social Responsibility

Throughout the most recent 20 years, CSR has been concentrated widely (Dobers and Halme, 2009; Lombardi et al., 2020; Maglio et al., 2020). Scholars have recognized that it has small and large-scale level consequences for a few significant components that clarify firm behavior (Vlachos et al., 2014). Likewise, Vlachos et al. (2014) have claimed that “*employees positively respond to CSR” to explain that “employees’ do get engaged in extra-role behaviors.*” In particular, workers’ judgment of their organization’s involvement in climate-related CSR practices emphatically impact their commitment and they embrace their firm’s CSR related projects by and large.

Corporate social responsibility strategy cultivates community-oriented values, sustainability values, and altruistic environmental values in the organizational culture (Afsar and Umrani, 2020). The work of Malik et al. (2020) proposes that workers’ impression of their firm’s inclusion in CSR practices connect with them to their firm’s CSR programs. Additionally, the workers’ PEB (a kind of extra-job practices) may likewise identifies with CSR. Consequently, it is imperative when and what CSR meant for workers’ commitment to PEB. The above hypothetical conversation upholds that pro-environmental and socially responsible firms are means to fascinate prospects expected representatives as they probably distinguish themselves with that sort of business.

Therefore, it is hypothesized that:

H5: *Pro-environmental behavior positively influences GCSR.*

#### Moderating Effect of Information Technology Capabilities

Nowadays, HRM practices and the ways organizations execute all their functions are largely determined by IT. IT has become an integral part of an organization contributing to unique advantages for organizations (Kengatharan, 2020). IT is an effective strategy for managing ecological issues. Because of expanding ecological mindfulness, the ecological repute and image during the recruitment assume an undeniably part in recruitment drives. Amongst the different forms of HRM functions, recruitment and selection derive embrace to pull in candidates and increment the number of qualified candidates (Jiang et al., 2012). Therefore, in analyzing the strategy adopted by the firm to receive and speak to its potential candidates are IT-based (Jiang et al., 2012). So, once employees get selected on the bases of green criterion and mindfulness then such HRM-driven green workforce will lessen costs and urge activities to improve the firm’s competitive position and development (Ray et al., 2005; Aral and Weill, 2007; del Brío et al., 2007). Information technology encourages the utilization of classified information and builds the knowledge integration capability of an organization and its employees (Benitez et al., 2018). Various contemplates have recognized the penetration of IT in all parts of the business and the significance of IT resources and capabilities in empowering business capacities that support firm to last and prosper (Ray et al., 2005; Aral and Weill, 2007).

Information technology and sustainability for businesses are ensured once strong networking and information exchange mechanisms are defined in an organization stance. The assortment of IT specialized skills through training encourages the arrangement of corporate information indexes and information organization (Benitez et al., 2018), which ultimately enhances the green consideration among worker’s behavior. Firms should guarantee that their HR regarding human capital and HR measures are employed to ensure their sustainable activities. In this way, managers should prepare and build up their representatives to develop human capital pitched toward sustainability (Youndt et al., 2004). It means following the sustainability viewpoint, the incorporation of IT skills and human resources are vital in empowering firms toward sustainability. The valuable integration and application of the IT capabilities provide feedback regarding workers’ performance on the job to evaluate their valuable contribution in true spirits; thus, they get rewarded and compensated accordingly. The lack of integration between the assimilated knowledge and process may not assist executives to interpret the available knowledge into eloquent info that can be exploited to achieve supreme benefits (Zahra and George, 2002). Consequently, incorporation of IT capabilities empowers firms involved in the CPEC and its employees to gain and absorb integrated knowledge. Hence, all IT capabilities help in devising an environment alongside HR resources that supports to profile green behavior of the employees. Therefore, we theorize the following:

H6: *IT capabilities positively affect green HRM practices (a) green recruitment and selection, (b) green training and development, and (c) green reward and compensation.*

## Methodology

### Measurements

We built up an investigation instrument utilizing the current studies and adjusted to the existing setting. The survey tool involved three extents; the principal segment incorporated research narrative; the subsequent segment had questions identified with demographics; the third section comprised questions related to the constructs in the research framework. In this study three green HRM practices were evaluated using the measurement scale of Jabbour et al. (2010); Saeed et al. (2019). This study measured the green recruitment and selection by employing nine items. We operational zed the three aspects of this recruitment and selection construct including green awareness, green employer branding, and green criteria to attract potential candidates.

Green training and development are adopted and contextualized within the existing study by using five items from Jabbour et al. (2010). GTD improves workers’ knowledge, abilities, and control while also increasing natural attention and preparing them to interact with the common environment It also permits them to improve their capacities and self-efficacy to address ecological issues in a powerful way that would assist representatives to assume accountable eco-friendly behavior in the form of pro-environmental behaviors.

We borrow the five items of green reward and compensation from Jabbour et al. (2010); Saeed et al. (2019). The adopted items deal with monetary or non-monetary rewards for attracting and retaining potential candidates. We altered it significantly to include monetary and non-monetary incentives such as tax incentives, companywide publicity, paid holidays, and voucher. We adopted a five items scale to measure pro-environmental psychology from Chou (2014) and slightly changed it to fit in the current setting. To measure pro-environmental behavior, we have utilized the eleven items from the studies of Kaiser et al. (2007); Robertson and Barling (2013), Kim et al. (2016). We used the statements to make employees familiar, encourage, and motivate, to perform environmental practices.

Six items were adapted to measure the IT capabilities from the work of Saraf et al. (2007); Muhanna and Dale Stoel (2010). The measures used for the CSR comprising 11 items to measure green CSR from Turker (2009). A five 5-point Likert scale was used to measure all items of the respective measures and adapted in the CPEC context. The members’ demographic qualities contained data about their sex, age, month-to-month pay, qualification, job titles, and length of experience.

The past investigations made were entirely in English in terms of the construct’s items. We somewhat changed these items to fit in the current setting. We followed Bracken and Barona (1991) methodology for back-translation to utilize the back-interpretation strategy and welcomed three phonetic educators from a neighborhood college to make an interpretation of the survey’s statements into the Urdu language. They assessed three distinctive deciphered adaptations, and in the wake of wiping out irregularities and inconsistencies built up the last English form with an agreement.

Further, two Urdu-talking senior academicians, who had insight into utilizing the back-interpretation strategy, deciphered the Urdu adaptation of poll once again into English. Also, they guaranteed the shortfall of glitches in importance, minor phrasing, and syntax issues through the validated measure. The researchers pretested the instrument by including 54 respondents who were not part of the conclusive review. They approved the rationality of the survey items without distinguishing any significant concern. Measurably, the after-effects of Cronbach’s alpha (α) acceptable i.e., value >0.70 got confirmed statistically which additionally affirmed the face and content validity of green recruitment and selection = 0.791, green training and development = 0.816, green reward and compensation = 0.773, = 0.759, pro-environmental psychological climate = 0.852, pro-environmental behavior = 0.831, and green corporates’ social responsibility = 0.794).

### Sampling and Data Collection

The firms involved in the CPEC project are considered a the population of this study. Because the CPEC is deemed to be a truly critical project with 62billion USD of investment to boost the socio-economic development in Pakistan. It is expected that the CPEC will give a raise to GDP of about 1.5% for the next 3 years. CPEC promises to be healthy for Pakistan’s economy and is likely to bring millions of job opportunities and avenues for the youth (Rathore et al., 2020). Moreover, this is a key opportunity for Pakistan to tackle the unemployment issues in Pakistan and give a ray of hope to youth in order to utilize expertise under one belt one road initiative is probably going to bring about a high number of employment openings going from 600,000 to 1,000,000 somewhere in the range of 2015 and 2030 with required exceptional and talented labor force (Rathore et al., 2020). In this way, significant consideration is needed to recognize issues identified with human resource development (HRD) that should be in accordance with the historic viewpoint of HRD. In like manner, the CPEC projects firms as populace for the research has been picked.

The gifted and organized labor force is continually reassuring for foreign investors and as per a Chinese authority, Pakistan doesn’t have the necessary labor force with top-notch abilities that are required concerning the advanced devices and methods and it is obvious factually that few representatives are being acquired from China to work in Pakistan (Rathore et al., 2020). That is why the officer’s grade employees of such firms working under the CPEC are the target sample for the underlying research. Infect, employees of these firms are capable enough of demonstrating green HRM practices to better establish green CSR while transforming the external knowledge to bring innovation through the successful implementation and completion of this very mega project. By following the guidelines of Lindner et al. (2001) about sample size, the sample size is 388.

In the current situation, the online overview was simple and helpful to gather data when contrasted with offline, for example, on-spot interventions, meetings, and examinations. In this way, the scholars accept that the online overview technique is proper for the current study.

The data collection took place between late August and early September 2020. The self-selected convenience sampling technique suggested by Kim and Qu (2020) was adopted for the current study for the selection of the sample. Ferber (1977) also endorsed that the questions items as part of the survey must be cohesive and applicable regarding the participants of the study, address the populace to truly serve up sample adequacy.

Accordingly, we put forth a concentrated effort while choosing a self-selected convenience sampling method to put an open end-question for screening the participants like; “have they ever been recruited and trained during a project. Eventually, 411 responses from participants were recorded. Among 411, 388 stood for the current investigation. We disposed of 23 responses because they were unclear (e.g., all scores as 1 or 5) and precluded (e.g., no related knowledge of enlistment, choice, training, and reward).

Since non-response bias and inclination are the fundamental challenges for studies using the survey method as a data collection system. However, the respondents were separated into two categories, late respondents and early respondents, according to the principles of Barriball and While (1999). To assess all variables, an independent sample t-test is being used. Initially, 250 responses were categorized as early, and later 138 responses were classified as late responses respectively (Karahanna and Straub, 1999). The results for non-response bias are shown in Table 1. The group means and SD for early and late responses are not significantly different, according to the findings.

**TABLE 1:** Results of early and late response.

	*N*	Mean		*SD*
	Statistic	Statistic	Std. Error	Statistic
**CSRG**	250	5.7825	0.04433	0.76776
	138	5.8504	0.07027	0.76003
**GRC**	250	5.6720	0.04931	0.85412
	138	5.7094	0.07606	0.82274
**GRD**	250	5.7208	0.05053	0.97526
	138	5.6624	0.09066	0.98059
**GTD**	250	5.4840	0.05118	0.88640
	138	5.3863	0.08455	0.91453
**ITC**	250	5.6791	0.04207	0.72869
	138	5.6620	0.07038	0.76124
**PEB**	250	5.7077	0.03886	0.77310
	138	5.7014	0.07047	0.76230
**PEPC**	250	5.6561	0.04274	0.74034
	138	5.6083	0.06367	0.68869

Further, to assess the normality, skewness and kurtosis tests were performed as suggested by Tabachnick and Fidell (2001) and the values were within the threshold level (±1, ±3). Table 2 specified that among 388 respondents, 7.,% were male and 25.3% were female. The largest age category was 31–40 years (85.4%), followed by 41–50 years (9.8%), and 30 years and above (4.9%). So far, as education level is concerned, 61 and 29% had graduate degrees and MPhil degrees, correspondingly. Regarding the executive positions, more than 57% of members had a place with a middle level of management, and 29.1 and 3.87% were from high-level and lower-level administration positions separately. Most of the respondents had a length of job experience of 17–21 years (35.3%), trailed by 11–16 years (29.6%), 22 years and over (18%), and 05–10 years (17%). About the greater part of the members was male (74.7.%).

**TABLE 2:** Tourists’ profile (*n* = 388).

Demographic information	Frequency	Percentage
***Gender***		
Male	289	74.7
Female	99	25.3
***Age (years)***		
30 years and above	68	17.5
31–40 years	273	70.4
41–50 years	35	9.0
51 and above	12	3.1
***Tenure (Years)***		
5–10 years	66	17.1
11–16 years	115	29.6
17–21 years	137	35.3
21 and above	70	18.0
***Education (Years)***		
12 and below	4	1.0
14	22	5.7
16	240	61.9
18 and above	114	29.4
***Management Level***		
Lower	113	29.6
Middle	225	57.4
Higher	50	13.0

## Data Analysis

For the current study, the researchers have employed the PLS-SEM technique for data analysis and results. Partial least squares-structural equation modeling (PLS-SEM) was chosen over CB SEM as the sample size was smaller than those of other related research (Ringle and Sarstedt, 2016). PLS-SEM is a composite-based estimation that has been frequently applied in management research to investigate structural equation models with latent variables at the same time. On the other hand, covariance based-structural equation modeling (CB-SEM) has indeed been commonly applied in the field of social science for decades, and it has been the ideal statistical method for affirming or dismissing theories thru the testing hypotheses nowadays, especially when the sample size is large, the data is normally distributed, as well as, notably, the model is properly stipulated (Hair et al., 2011). PLS is a soft modeling approach to SEM with no assumptions about data distribution (Vinzi et al., 2010). It’s worth noting that SmartPLS believes the indicators are reflecting, exhibiting directional arrows away from the blue-color observed variables when it develops the model as in the underlying study. Unless the indicators are deeply connected and interchangeable, they are reflective and therefore should be thoroughly evaluated for reliability and validity (Haenlein and Kaplan, 2004; Hair et al., 2013). PLS path modeling can be done using a variety of applications (Wong, 2013), however, SmartPLS has gained favor in recent years (Ringle and Sarstedt, 2016). Version 3 of this software includes additional capabilities that allow researchers to automate various statistical operations that were previously only possible to do manually.

### Assessment of the Measurement Model

Following (Hair et al., 2017), the researchers looked at the outer model’s reliability and validity. Internal consistency and convergent reliability were tested using the standardized factor loadings (SFL), composite reliability (CR), and average variance extracted (AVE). The results indicated in, CR, AVE, and inner variance inflation factor (VIF) values over 0.708, 0.70, and 0.50, respectively, indicating acceptable reliability and convergent validity (Hair et al., 2017) as exhibited in Table 3. Moreover, discriminant validity was proven using the method proposed by Fornell and Larcker (1981), wherein the correlation among observed variables is always less than the square root of AVE. In addition, the advanced method of the Heterotrait-Monotrait Ratio Inference (HTMT) criterion test is being used to confirm discriminant validity (Henseler et al., 2016). The discriminant validity is present in accordance with the previous test including HTMT values with confidence intervals (CI: 90%) (Table 3). Lastly, model fit got established through certain results of χ^2^ 5.1273.708, SRMR 5.071, RMS theta 5.0051, and NFI 5.820 (Hair et al., 2017). Table 4 presents the outer loading for each item.

**TABLE 3:** Goodness of fit and average variance extracted (AVE).

	Cronbach’s alpha	rho_A	Composite reliability	Average variance extracted	Inner VIF
**CSRG**	0.945	0.950	0.953	0.649	
**GRC**	0.802	0.802	0.871	0.628	1.730
**GRS**	0.954	0.956	0.960	0.729	2.270
**GTD**	0.898	0.905	0.925	0.711	2.070
**ITC**	1.000	1.000	1.000	1.000	1.771
**PEB**	0.964	0.965	0.968	0.736	2.371
**PEPC**	0.886	0.893	0.917	0.690	2.371

**TABLE 4:** Factor loading of all items.

Constructs	Items	Statements	FL
**Green corporate social responsibility (CSRG)**	CSRg1	The corporate ethics (ethical behavior in interactions with public official, politicians and other enterprises) is prevalent in my organization.	0.728
	CSRg2	My organization makes investment to create a better life for future generation.	0.779
	CSRg3	My organization implements special programs to minimize its negative impact on the natural environment.	0.766
	CSRg4	My organization Companies in your country My organization encourage workers to volunteer for social causes and have incentives that facilitate that involvement.	0.754
	CSRg6	My organization exercises corporate codes of conduct and other aspects of corporate social responsibility.	0.719
	CSRg7	My Organization consider cleaner production, material flow management, waste reduction and recycling, and life cycle management of products.	0.751
	CSRg8	My Organization adhere to environmental management systems such as ISO 14000.	0.849
	CSRg9	My organization now considers long-term factors such as global climate change and other environmental risks a part of business planning.	0.869
	CSRg10	My organization involves in Corporate environmental reporting.	0.877
	CSRg11	My organization participates in activities, which aim to protect and improve the quality of the natural environment.	0.792
**Green Recruitment and selection (GRS)**	GRS1	In my organization job description specification includes environmental concerns.	0.709
	GRS2	My organization selects applicants who are sufficiently aware of greening to fill job vacancies.	0.826
	GRS3	In my organization recruitment messages include environmental behavior/commitment criteria.	0.851
	GRS4	In my organization job positions are designed that focus exclusively on environmental management aspects of the organizations.	0.884
	GRD6	My organization includes environmental criteria in the recruitment messages.	0.905
	GRS7	My organization expresses the preference to recruit candidates who have competency and attitudes to participate in corporate environmental management initiatives too in the recruitment message.	0.871
	GRS8	My organization considers candidates’ environmental concern and interest as selection criteria.	0.900
	GRS9	My organization when interviewing candidates or evaluating them for selection, asks environment-related questions.	0.893
**Green training and development (GTD)**	GTD2	My organization takes into account the needs of environmental issues when training requirement are analyzed.	0.899
	GTD4	My Organization provides training to learn or adapt environmentally friendly best practices (e.g., reducing long-distance business travel and recycling).	0.914
	GTD5	My organization is applying job rotation to train green managers of the future.	0.714
**Green reward and Compensation (GRC)**	GRC1	My organization offers a non-monetary and monetary rewards based on the environmental achievements.	0.856
	GRC2	In my organization environmental performance is recognized publicly.	0.786
	GRC3	My organization is Introducing rewards for innovative environmental initiative/performance.	0.833
	GRC4	The corporate ethics (ethical behavior in interactions with public official, politicians and other enterprises) is prevalent in my organization.	0.874
**Pro-environmental Behavior (PEB)**	PEB1	I make suggestions and bring new ideas about environmentally friendly practices to environmental committees.	0.824
	PEB2	I share my knowledge about the environment with co-workers.	0.891
	PEB4	At work, I question practices that are likely to hurt the environment.	0.881
	PEB6	At work, I perform environmental tasks that are not required by my company.	0.863
	PEB7	At work, I avoid wasting resources such as electricity or water.	0.786
	PEB8	At work, I take stairs instead of elevators to save energy.	0.862
	PEB9	At work, I turn off lights when out of office.	0.735
	PEB 11	At work, I recycle (e.g., paper, cans, batteries, oil).	0.851
**Pro-environmental psychological climate (PEPC)**	PEPC2	Saving energy is important in my organization.	0.900
	PEPC3	Saving water is pivotal in my organization.	0.845
	PEPC4	In my organization reduction in the use of disposable products is critical.	0.936
	PEPC5	Waste reduction and the control of harmful materials mean a lot to this organization	0.850
	PEPC2	Saving energy is important in my organization.	0.900
**Information technology capabilities (ITC)**	ITC1	IT systems for new product development projects.	0.785
	ITC3	IT systems for facilitating technology knowledge creation.	0.871
	ITC4	IT systems for facilitating market knowledge creation.	0.755
	ITC5	IT systems for internal communication (e.g., across different departments, across different levels of the organization).	0.765
	ITC6	IT systems for external communication (e.g., suppliers, customers, channel members	0.736

### Heterotrait-Monotrait Ratio

Table 4 shows that the HTMT ratios are less than the 0.85 criterion, indicating that the reflective construct’s discriminant validity is supported (Petter et al., 2007). The Cronbach alpha coefficient test was used to determine the questionnaire’s internal consistency, with an appropriate given threshold value of 0.70. The inconclusive values of skewness and kurtosis which were ≥1.96 have confirmed that the data was not normally distributed. Furthermore, for discriminant validity Heterotrait-Monotrait (HTMT) ratio values were determined. According to Petter et al. (2007), HTMT values with a threshold of 0.85, indicated that the results were below the permissible range, implying the discriminant validity as shown in Table 5.

**TABLE 5:** Heterotrait-Monotrait (HTMT) ratio.

	1	2	3	4	5	6
**CSRG**						
**GRC**	0.654					
**GRS**	0.588	0.703				
**GTD**	0.603	0.670	0.748			
**ITC**	0.645	0.546	0.668	0.543		
**PEB**	0.531	0.699	0.503	0.545	0.588	
**PEPC**	0.549	0.615	0.562	0.592	0.575	0.823

### Common Method Bias

According to the definition proposed by Podsakoff et al. (2003), CMB is “the variance that is attributed to the method of measurement rather than to the construct the measurement represents” and in general deals with measurement error. When CMB is sufficiently high, inaccurate deductions regarding supposed correlations might well be drowned, much like other sorts of measurement errors. There are a variety of great methods and approaches to minimizing CMB. These techniques include preventive steps aimed at reducing CMB prior to data collection and statistical approaches for detecting and regulating or reducing findings’ potential bias during the data processing phase (Chin et al., 2012). Podsakoff et al. (2013), suggested different strategies for significantly reducing and monitoring CMB, including (1) obtaining predictor (IV) and criterion (DV) indicators from diverse sources, (2) measurement methodological separation, (3) neutralize the order of inquiries, (4) seeking to protect the participants’ privacy, and (5) using the reverse source item. Normally, similar precautions are implemented all through the study’s design phase and the survey instrument development phase.

### Structural Model and Hypotheses Results

**FIGURE 2:**
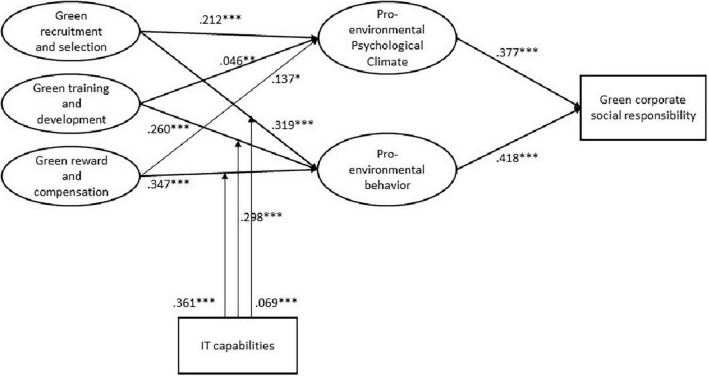
Results of the hypotheses.

After the reasonable results of the estimation model, path analysis was performed for the structural model to test the hypothesis of the study. Figure 1 demonstrates the results for the structural model. In order to establish the o\path significance, bootstrapping with 1,000 samples was performed. The findings reveal that four constructs account for 30.6 percent of the variation in CSRG and that this study model accounts for 23 percent and 29.3 percent of the variance in PEPC and PEB, respectively. All of the study’s hypotheses were confirmed, as shown in Table 6. GRS had a significant impact on PEPC (=0.212, *p* 0.000). Similarly, GRS has a significant impact on PEB (=0.319, *p* 0.000), confirming the hypotheses H1 and H2. As per the results of H3, GTD does have an insignificant positive effect on PEPC (=0.046, *p* 0.000). In addition, PEB is significantly influenced by GTD (=0.260, *p* 0.000), confirming H4. GRC does have a negligible significant effect on PEPC (=0.137, *p* 0.000), according to the results of hypothesis H4. In addition, GRC has a substantial significantly positive effect on PEB (=0.347, *p* 0.000), confirming H5. PEPC has a substantial positive impact on PEB (=0.182, *p* 0.001), as per H6. The H7 and H8 present the direct relationships between the mediator and dependent variables and show that PEPC and PEB have a significant positive effect on CSRG (β = 0.337, *p* < 0.000) and (β = 0.418, *p* < 0.000) respectively. PEPC and PEB exhibit a significant positive effects on CSRG (=0.337, p 0.000) and (=0.418, p 0.000), in both, thus confirming H7 and H8. The path coefficient (β) value and the significant values of all direct relations are shown in Table 6. Moreover, IT capabilities significantly moderates the association between green recruitment and selection and pro-environmental behavior (β = 0.361, *t* = 7.520, [CI:0.2669,0.4551], *p* < 0.000), green training and development and pro-environmental behavior (β = 0.298, *t* = 6.081, [CI:0.2020,0.3940], *p* < 0.000), and green reward and compensation and pro-environmental behavior (β = 0.069, *t* = 0.750, [CI:–0.1113,0.2493], *p* < 0.000) as demonstrated in Figure 2 and Table 6. Hence H6 (a), (b), and (c) were accepted.

**TABLE 6:** Results of the hypotheses.

	Original sample (O)	Sample mean (M)	Standard deviation (STDEV)	t Statistics (| O/STDEV|)	*P*-value
**GRC → PEPC**	0.137	0.145	0.058	2.353	*
**GRS → PEPC**	0.212	0.207	0.056	3.819	***
**GTD → PEPC**	0.046	0.047	0.043	1.059	**
**GRS → PEB**	0.319	0.321	0.069	4.623	***
**GTD → PEB**	0.260	0.297	0.083	3.132	***
**GRC → PEB**	0.347	0.332	0.079	4.392	***
**PEB → CSRG**	0.418	0.419	0.083	5.034	***
**PEPC → CSRG**	0.377	0.377	0.085	4.433	***
**IT × GRC → PEB**	0.361	0.298	0.048	7.520	***
**IT × GRS → PEB**	0.069	0.319	0.092	0.750	***
**IT × GTD → PEB**	0.298	0.342	0.049	6.081	***

### Goodness of Fit

Partial least squares-structural equation (PLS-SEM) does not provide general Goodness of Fit (GoF) data because presenting PLS routes does not improve any international numeric capability and often falls short on a listing that could provide the users with a worldwide Model acceptance. As a result, evaluating the R^2^ values is a crucial tool for assessing the model’s explanatory ability (Chin et al., 2012). The goodness of fit demonstrates an operative solution to this challenge; it could be used as a global index for validating the PLS model.

The goodness of fit shows an operational answer for this issue; it very well might be implied as a list for the approval of the PLS model internationally. The arithmetic mean of average commonalities and the average R^2^ are used to assess the goodness of fit. For the predictor variables, the R^2^ estimate is not possible. The quality of fit is calculated as follows, GoF = √0.690*0.276 = √0.191 = 0.437. The goodness of fit estimates in this study is 0.437, indicating that the PLS model is fit. It demonstrates that the hypothetical model can take into account 43.70 percent of a possible fit so the model is satisfactory.

## Discussion and Conclusion

Megaprojects have broad and major societal responsibility throughout their existence. Megaprojects, particularly infrastructure improvements, play critical roles in the financial and social turn of events, and their social responsibility and sustainability have recently drawn unending attention. Megaprojects’ prolonged lifecycles and diverse partners have created remarkable challenges for managing monetary, social, and environmental issues. The CPEC project has grown in importance not only in Pakistan but also globally. As a result, the researchers were inspired to investigate the influence of GHRM practices on green CSR in the context of CPEC. Using stakeholder theory, we developed and rigorously validated the suggested study model for evaluating the role of green HRM practices in predicting green CSR among CPEC enterprises.

This framework covered the CPEC enterprises’ predictors (green recruiting and selection, green training and development, and green reward and remuneration) and outcomes (green CSR). We contribute to the literature by including pro-environmental psychological climate as a first outcome in the correlation of antecedents and pro-environmental behavior, as well as the role of pro-environmental behavior in the correlation of antecedents and effects. The findings revealed that green HRM practices have a significant impact on green CSR. Furthermore, the postulated moderators’ pro-environmental psychological climate and pro-environmental behavior had a positive effect on green CSR. Similarly, the postulated moderator IT capabilities had a positive effect on green CSR. In this vein, our findings provide hypothetical and administrative implications to address the academic and administrative inquiries. In this approach, our findings provide hypothetical and administrative implications to address academic and administrative queries.

### Theoretical Implications

Our research makes several key contributions to organizational theory. In a variety of ways, the current inquiry results present numerous significant commitments to the assemblage of the firm’s conduct, ethics, knowledge management, and information technology. First, environmental concerns have enlarged the company’s role in preserving the environment (Islam et al., 2019). Firms have turned to green activities in response to increased demands, leading to the recognition of green HRM practices (Islam et al., 2020), despite the lack of empirical study on the environmental effects (Islam et al., 2020).

Our study’s findings indicate that green recruitment and selection (GRS) and green reward and compensation (GRC) methods are the most important. Yuriev et al. (2018); Yusliza et al. (2019) hold an opposing viewpoint to our study’s findings, which demonstrate that the suitable candidates following this GRS procedure are capable of establishing their views and acting accordingly and organizational behavior.

Nonetheless, the literature has demonstrated a positive causal relationship between green training and green employee behavior (Teixeira et al., 2012; Islam et al., 2019). Training research has also resulted in increased workload (Oppenauer and Van De Voorde, 2018), time constraints, and stress. Our data show that PEB has a detrimental influence, which is consistent with the findings of the study (Yuriev et al., 2018). On the contrary, Fernández et al. (2003), advocated for environment-oriented training to raise employees’ awareness, knowledge, and skills in order to develop a green climate. Since our analysis confirms the successful role of GTD in creating such an atmosphere, which is consistent with the study of Zoogah (2011), it stimulates each personnel to embrace eco-friendly programs while verifying our proposed theory. Green reward and compensation systems, according to Jabbour et al. (2013), aim to align green performance and activities accepted in the firm. According to experts (Daily and Huang, 2001; Jackson et al., 2011), incentives encourage employees to actively participate in organizational green functions. This opinion was validated by our research, which adds to the body of sustainable HRM practices in which green GRC within a company influences the working environment and pro-environmental behavior among employees, as contented by Dumont et al. (2017).

According to our findings, the significance of the environment in creating a strong work environment, which is also supported by Wehrmeyer and Parker (1996). Similarly, our study stresses that newly selected personnel will develop positive pro-environmental behavior after joining respective firms. Similarly, green climate assists employees in demonstrating such green activities that truly provide support to our H1, as studied by del Brío et al. (2007). This significant association contributes to the body of knowledge on GHRM practices in shaping organizational culture to support green CSR practices and instilling green behavior is crucial.

In contrast to other studies (Afsar and Umrani, 2020) our study has further shown the substantial importance of PEB in promoting green corporate social obligations in the organization. However, our study’s findings showing a positive relationship between PEB and Green CSR are consistent with the findings of Chaudhary (2020). As a result, our H4 and H5 hypotheses are confirmed.

It is regarded as the fourth factor of production and has played an important role in developing, designing, and implementing organizational human resource strategies (Cooper et al., 2019). Information technology encourages the distribution of classified information while also developing an organization’s and its workers’ knowledge integration capability (Benitez et al., 2016). Indeed, our study has verified H6, where the positive function of IT skills in supporting green work behavior has been demonstrated, and this is consistent with the study done by Ojo and Raman (2019). Furthermore, IT capabilities can strengthen an organization’s green image, verifying the business’s long-term viability. As a result, our research helps firms establish and design their performance management systems and sustainability capabilities.

Finally, the “performance paradox” of megaproject execution is consistent and evident in various configurations. Keeping this in mind, we chose social responsibility, which has drawn in far-reaching thought but has not been broken down effectively and profoundly enough in managing megaprojects that are even debated by researchers (Ma et al., 2017). Furthermore, previous research reveals that megaprojects in developing countries largely ignore the importance of human capital and human resource development in completing megaprojects successfully (Rathore et al., 2020). Pakistan has emphasized unskilled skilled labor (McLean, 2006). However, our study supports the critical significance of GHRM practices and intends to offer value in this area while prioritizing green HRM practices for the seamless execution of this project.

## Practical Implications

The article includes managers with a variety of practical suggestions for improving green CSR. For a long-term manageable development, CSR initiatives must first be integrated within the firm’s formal social goals. A growing number of studies like (Saeed et al., 2019; Hameed et al., 2020), revealed that in order for firms to efficiently and successfully implement green policies, green HRM practices should be adopted. This will also assist HR supervisors/head trackers in identifying and recruiting potential helpful ecological workers. As a result, employees of CPEC-affiliated enterprises must embrace green HRM practices as predictors of green CSR.

The need of the hour is for policymakers to focus on HRD while laying more foundations, all else being equal (professional and expert), which may support the availability of a certified labor force for CPEC. Furthermore, if businesses want to enable individuals and organizations as a whole to engage in green initiatives, they may benefit from implementing green HRM methods. Executives must include CSR activities into company HR policies as well as review perceptions of employees for the firm’s CSR efforts on a routine basis. Businesses must provide training and development opportunities for their employees in order for them to better comprehend green principles (Hameed et al., 2020). Managers of some Bangladeshi enterprises, such as Asian apparel and Mahmud jeans ltd., are examples (Shah Ridwan Chowdhury and Asaduzzaman, 2017).

Companies must incorporate environmentally friendly job descriptions and job design into their recruitment and selection procedures. Interviews can contain environmental questions to determine the candidates’ level of awareness, concern, understanding, and dedication to the environment. Finally, firms should adequately assess their employees’ green behavior (Dumont et al., 2017). Firms, for example, can disseminate such activities through formal reports, official websites, emails, seminars, TV ads, or publications to make employees aware of the firm’s socially responsible initiatives. Furthermore, organizations can adopt such ideas into their culture through successful methods.

Workers need to be incentivized to come up with innovative solutions to promote low-energy work patterns. The hypothesized model under consideration in this inquiry is intended to serve as a reference for top/middle level managers of the individual enterprises functioning under CPEC in order to better identify and evaluate the company’s condition. It may contribute to the enhancement of important dynamic and strategic configuration measures for the effective and timely completion of CPEC projects. The framework ensures the expansion of such channels and technology that have flourished in the firm’s core green atmosphere and green conduct. Firms might also use their resources to initiate proactive measures to reduce environmental damage. Finally, because of the interaction influence of IT capabilities in creating representative pro-environmental behavior, workers should be prepared to best practice green activities, invigorate eco-friendly programs, and provide help to participate in ecological practices. IT capabilities can assist the company in planning, communicating, preparing, and executing such HR operations.

## Limitations and Future Research Directions

The understudy research findings and outcomes must be viewed in the perspective of the its limitations as well. Principally, instead of an objective administrator or peer judgments, perceptions, or documented statistics, individuals self-reported overall green CSR. Although this may appear on the surface that self-report measures are skewed. Gifford and Nilsson (2014), demonstrated through meta-analysis that self-reported and target green practices can be compared (=0.46), proposing that employees can reasonably survey their green direct. We are confident that the conclusions of this study will not be influenced by frequent technique bias discovered through construct validity tests. Second, a cross-sectional approach was used to collect data. Consequently, determining causation in the study’s hypotheses is challenging. For additional investigation, a longitudinal research strategy is advised. Third, the assessment of green HRM practices was centered on the subjective reactions of employees. Similarly, we need to be aware of the possible distinction between discernment and truth in a firm’s green HRM practices related to GCSR.

Finally, the editing research framework may be validated using characteristics such as corporate culture, organizational citizenship behavior, and sustainability. Future research should look into different variables as modifiers, such as green self-efficacy and environmental consciousness. Green commitment, work happiness, and a green lifestyle, on the other hand, are suggested mediators for future researchers.

## Data Availability Statement

The original contributions presented in the study are included in the article/supplementary material, further inquiries can be directed to the corresponding author/s.

## Author Contributions

RH contributed to the conceptualization and writing the first draft of the research. MS and AM contributed to visualizing and supervising the research. All authors who contributed to the manuscript read and approved the submitted version.

## Conflict of Interest

The authors declare that the research was conducted in the absence of any commercial or financial relationships that could be construed as a potential conflict of interest.

## Publisher’s Note

All claims expressed in this article are solely those of the authors and do not necessarily represent those of their affiliated organizations, or those of the publisher, the editors and the reviewers. Any product that may be evaluated in this article, or claim that may be made by its manufacturer, is not guaranteed or endorsed by the publisher.
